# Esophageal Injury During Transesophageal Echocardiography-Guided Left Atrial Appendage Occlusion: A Narrative Review

**DOI:** 10.3390/diagnostics16213631

**Published:** 2026-11-03

**Authors:** Jonatan Vuković, Tina Bečić, Josipa Radić, Mislav Radić, Damir Fabijanić, Ivana Jukić

**Affiliations:** 1 Department of Internal Medicine, Division of Gastroenterology, University Hospital of Split, 21000 Split, Croatia; jonatan.vukovic@mefst.hr; 2 Department of Internal Medicine, School of Medicine, University of Split, 21000 Split, Croatia; josiparadic1973@gmail.com (J.R.); mislavradic@gmail.com (M.R.); 3 Department of Cardiovascular Diseases, University Hospital of Split, 21000 Split, Croatia; tina.becic@gmail.com (T.B.); damirfabijanic62@gmail.com (D.F.); 4 Department of Internal Medicine, Division of Nephrology and Haemodialysis, University Hospital of Split, 21000 Split, Croatia; 5 Department of Internal Medicine, Division of Rheumatology, Allergology and Clinical Immunology, University Hospital of Split, 21000 Split, Croatia; 6 Department of Clinical Propedeutics, School of Medicine, University of Split, 21000 Split, Croatia; 7 Faculty of Health Sciences, University of Split, 21000 Split, Croatia

**Keywords:** left atrial appendage occlusion, transesophageal echocardiography, esophageal injury, gastrointestinal complications, intracardiac echocardiography, cardiac computed tomography, multimodality imaging

## Abstract

Transesophageal echocardiography (TEE) remains an established imaging modality for guiding percutaneous left atrial appendage occlusion (LAAO), providing real-time visualization during transseptal puncture, device deployment, and procedural assessment. However, esophageal instrumentation and prolonged probe manipulation may result in gastrointestinal injury, particularly in patients with pre-existing esophageal abnormalities or increased bleeding risk. This narrative review summarizes the available evidence regarding the mechanisms, risk factors, clinical manifestations, diagnosis, management, and prevention of TEE-associated esophageal injury during LAAO while examining alternative imaging strategies. A literature search was conducted in PubMed, Scopus, Web of Science, and the Cochrane Library, supplemented by targeted searches of broader structural heart intervention literature. Although clinically recognized TEE-associated gastrointestinal complications are uncommon, studies employing systematic endoscopic surveillance have identified substantially higher rates of subclinical mucosal injury. Reported manifestations range from transient dysphagia and superficial mucosal lesions to gastrointestinal bleeding, intramural esophageal hematoma, perforation, and fistula formation. LAAO-specific registry and administrative datasets report gastrointestinal bleeding rates ranging from approximately 0.2% to 8.2%, but heterogeneous outcome definitions, ascertainment methods, and the absence of systematic endoscopic verification prevent these findings from being interpreted as rates of TEE-associated esophageal injury. Nevertheless, limited LAAO-specific evidence, heterogeneous outcome definitions, and differences in surveillance strategies preclude reliable estimation of procedure-specific esophageal injury incidence. Intracardiac echocardiography eliminates esophageal instrumentation but carries a distinct procedural risk profile, whereas cardiac computed tomography (CCT) provides complementary opportunities for preprocedural planning and postprocedural surveillance. Careful preprocedural assessment, judicious probe manipulation, early recognition of complications, and individualized imaging selection are important considerations. Prospective studies incorporating standardized esophageal injury endpoints are needed to establish the true clinical burden and guide preventive strategies.

## 1. Introduction

Atrial fibrillation (AF) is the most common sustained cardiac arrhythmia and is associated with a substantial increase in the risk of ischemic stroke and systemic thromboembolism. Because the left atrial appendage (LAA) represents the predominant site of thrombus formation in patients with non-valvular AF, oral anticoagulation remains the cornerstone of stroke prevention. However, a clinically relevant proportion of patients have contraindications to long-term anticoagulation or remain at high risk of major bleeding despite appropriate therapy. In this setting, percutaneous left atrial appendage occlusion (LAAO) has emerged as an established non-pharmacological strategy for stroke prevention, particularly in selected patients in whom long-term oral anticoagulation is unsuitable or associated with excessive bleeding risk [1,2]. Accurate imaging is fundamental throughout the LAAO pathway, including pre-procedural assessment of LAA anatomy and exclusion of intracardiac thrombus, intraprocedural guidance of transseptal puncture and device deployment, and post-procedural assessment of device position, peri-device leak, and device-related thrombus. Transesophageal echocardiography (TEE) has historically been the most widely used intraprocedural imaging modality because it provides high-resolution, real-time multiplanar visualization of the LAA and surrounding structures. Contemporary practice, however, increasingly incorporates intracardiac echocardiography (ICE) and cardiac computed tomography (CCT) within multimodality imaging pathways [2,3,4,5]. Despite its established role, TEE is a semi-invasive procedure requiring insertion and repeated manipulation of an ultrasound probe within the upper gastrointestinal tract and is therefore not devoid of procedure-related complications [6,7,8]. Clinically recognized gastrointestinal injury following TEE is uncommon, but the reported spectrum ranges from transient dysphagia and odynophagia to mucosal laceration, gastrointestinal bleeding, intramural esophageal hematoma, and, rarely, esophageal perforation [6,9,10,11]. While clinically reported TEE-associated gastrointestinal adverse events are relatively uncommon [9], studies incorporating systematic endoscopic assessment have identified a substantially broader spectrum of mucosal abnormalities, including lesions that may remain clinically unrecognized [10,12,13].

Minor lesions may remain asymptomatic or cause only transient symptoms and therefore escape detection when diagnostic evaluation is symptom-driven. Conversely, endoscopically detected petechiae, ecchymoses, or superficial mucosal lesions should not be equated with clinically significant complications. Importantly, upper gastrointestinal injury should not automatically be interpreted as esophageal injury specifically [9,10,14,15,16]. Additional evidence from studies incorporating systematic endoscopic assessment, including those by Omuro et al. and Kuecken et al., further supports the distinction between clinically recognized complications and endoscopically detected mucosal injury [12,13]. However, differences in patient populations, procedural TEE exposure, and lesion definitions limit direct comparisons and prevent reliable extrapolation of these findings to LAAO. These considerations may be particularly relevant to patients undergoing LAAO. This population is typically older, has a substantial comorbidity burden, and frequently includes patients in whom long-term anticoagulation is contraindicated or associated with an unfavorable bleeding-risk profile [1,2]. Previous gastrointestinal bleeding is especially relevant and should not be regarded as a uniform phenotype because bleeding source, severity, recurrence pattern, endoscopic findings, and definitive treatment of the underlying lesion may materially influence subsequent hemorrhagic vulnerability [17]. At the same time, intraprocedural TEE may require repeated probe manipulation throughout transseptal puncture, device sizing, deployment, stability assessment, and evaluation for peri-device leak or procedural complications [2,3,5]. Direct LAAO-specific evidence regarding TEE-associated esophageal injury remains limited. Available observational studies have primarily reported broader procedural or gastrointestinal adverse-event outcomes, while individual case reports have documented rare but potentially severe esophageal complications [18,19,20,21]. However, these findings do not permit reliable estimation of the incidence of esophageal injury specifically attributable to TEE during LAAO. Furthermore, evidence derived from mixed structural heart intervention populations cannot be directly extrapolated to LAAO because of differences in patient characteristics, procedural exposure, and outcome definitions. The increasing adoption of ICE further changes the clinical context in which these complications should be considered. Contemporary meta-analyses have demonstrated broadly comparable procedural outcomes between ICE- and TEE-guided LAAO, although differences in specific complication profiles have been reported [22,23,24]. These findings are complemented by observational studies and registry-based comparisons [25,26]. Importantly, the available meta-analyses did not provide pooled estimates of esophageal or upper gastrointestinal injury, limiting their ability to quantify the potential safety advantage of avoiding esophageal instrumentation. Moreover, ICE has been associated with higher rates of certain complications, particularly pericardial effusion and residual iatrogenic atrial septal defect, in some comparative analyses [22,23,24]. Previous reviews have addressed complementary aspects of echocardiographic guidance and TEE-associated complications. Becic et al. systematically evaluated TEE and ICE guidance during LAAO, with particular emphasis on procedural outcomes in high-risk populations, including patients with chronic liver disease and previous gastrointestinal bleeding [27]. Separately, De Luca et al. reviewed TEE-associated complications across cardiac surgery and transcatheter structural heart interventions, focusing on predisposing factors and preventive strategies [28]. However, neither review specifically focused on the clinical and methodological challenges of identifying and interpreting TEE-associated esophageal injury during LAAO. Despite growing recognition of TEE-associated gastrointestinal complications, important gaps remain regarding the incidence, clinical significance, and determinants of esophageal injury specifically during LAAO. The available evidence is limited and heterogeneous, comprising LAAO-specific observational studies, individual case reports, and findings from broader cardiac intervention populations with differing outcome definitions and surveillance strategies. Against this background, the present narrative review aims to critically synthesize the available evidence regarding the spectrum, potential mechanisms, risk factors, clinical presentation, diagnosis, management, and prevention of TEE-associated esophageal injury during LAAO. Particular emphasis is placed on distinguishing direct esophageal injury from broader gastrointestinal adverse events and assessing the potential role of alternative imaging strategies in patients at increased esophageal risk.

## 2. Materials and Methods

A narrative review of the literature was conducted to identify and synthesize evidence regarding esophageal and upper gastrointestinal injury associated with TEE during LAAO, with additional consideration of alternative imaging strategies in patients at increased esophageal risk. The literature search was performed in PubMed, Scopus, Web of Science Core Collection, and the Cochrane Library from database inception to 26 September 2026.

The primary focused search strategy combined terms related to LAAO, TEE, and esophageal or upper gastrointestinal injury as follows: (“left atrial appendage occlusion” OR “left atrial appendage closure” OR LAAO OR LAAC) AND (“transesophageal echocardiography” OR “transoesophageal echocardiography” OR TEE OR TOE) AND (esophag* OR oesophag* OR “upper gastrointestinal” OR dysphagia OR odynophagia OR hematoma OR perforation OR laceration OR “mucosal injury” OR “probe injury” OR “TEE-related complication”). Because direct evidence specifically addressing esophageal injury during TEE-guided LAAO is limited, the focused search was complemented by targeted searches addressing TEE-associated gastrointestinal and esophageal complications in broader structural heart and perioperative populations. Supplementary searches also included terms related to intracardiac echocardiography (ICE), miniaturized or micro-TEE, CCT, multimodality imaging, procedural sedation, and alternative imaging guidance for LAAO. Reference lists of relevant original studies, systematic reviews, clinical guidelines, expert consensus documents, and selected case reports were additionally screened to identify potentially relevant publications not captured by the primary search strategy. The retrieved publications were examined for their relevance to the predefined clinical themes, with particular attention to studies reporting LAAO-specific gastrointestinal or esophageal outcomes. Studies involving mixed structural heart intervention populations were reviewed to determine whether separate LAAO subgroup data were available. When such data were not reported, the findings were used only as contextual evidence. Particular attention was given to study design, methods of outcome ascertainment, and the distinction between clinically recognized complications and systematically detected mucosal lesions. As this was a narrative review, the literature was selected for clinical relevance rather than through a formal systematic study-selection process.

Evidence was considered relevant when it addressed (1) the incidence or spectrum of TEE-associated esophageal or upper gastrointestinal injury; (2) mechanisms and procedural determinants of injury; (3) patient-related susceptibility, including pre-existing esophageal or upper gastrointestinal disease; (4) clinical presentation, diagnosis, or management of TEE-related injury; or (5) alternative imaging strategies capable of reducing or eliminating esophageal instrumentation during LAAO. Priority was given to LAAO-specific studies whenever available. Evidence from mixed structural heart, perioperative, and general TEE populations was used primarily to provide mechanistic, safety, and risk-context information and was not interpreted as providing LAAO-specific incidence estimates.

Given the substantial heterogeneity in study populations, procedural settings, definitions of gastrointestinal injury, surveillance intensity, and imaging strategies, no quantitative pooling was performed. The evidence was therefore synthesized narratively according to major clinical themes, including the burden and interpretation of available evidence, mechanisms of injury, patient vulnerability, clinical presentation and management, prevention, and the role of alternative imaging modalities. Given the narrative design of the review, formal risk-of-bias assessment and protocol registration were not performed.

The focused literature search identified seven publications reporting separately identifiable LAAO-specific data relevant to esophageal injury or gastrointestinal complications. These comprised five observational studies, including four retrospective studies and one prospective case series, and two case reports [18,19,20,21,29,30,31]. Two observational studies included mixed structural heart intervention populations but provided separately extractable LAAO subgroup results. Studies without separately reported LAAO outcomes were considered contextual evidence and were not included in the LAAO-specific evidence summary. Importantly, none of the seven publications systematically assessed subclinical esophageal mucosal injury using routine postprocedural endoscopy. Furthermore, the available registry-based studies did not establish whether the reported esophageal injuries or gastrointestinal bleeding events were directly attributable to TEE instrumentation.

## 3. Evidence Synthesis

### 3.1. Clinical Burden and Interpretation of the Available Evidence

The principal message emerging from the available literature is not a reliable numerical estimate of esophageal injury during LAAO, but rather the substantial uncertainty surrounding its true frequency. Studies relying on clinically recognized adverse events generally report low frequencies of gastrointestinal complications following TEE [9,14,15], whereas investigations incorporating systematic endoscopic assessment have identified a considerably greater burden of mucosal abnormalities in selected populations [10,12,13]. Gangwani et al. reported an overall gastrointestinal adverse-event rate of 0.51% across 26 studies involving 55,319 patients [9]. In contrast, Freitas-Ferraz et al. identified new upper gastrointestinal injury in 43 of 50 patients (86%) undergoing TEE-guided structural cardiac interventions, including complex lesions in 20 patients (40%) [10]. Although 24 patients in this cohort underwent LAA closure, the overall endoscopic injury rates should not be interpreted as LAAO-specific estimates. Furthermore, these studies used fundamentally different outcome definitions and surveillance strategies, and their findings should not be treated as competing estimates of the same event. Additional evidence from studies incorporating systematic endoscopic assessment supports the distinction between clinically recognized complications and mucosal lesions detected through active surveillance. Omuro et al. evaluated 262 patients undergoing pulmonary vein isolation for atrial fibrillation following preprocedural TEE and identified TEE-associated esophageal mucosal injury in 16 patients (6.1%) by postprocedural endoscopy [12]. In a separate cohort of 40 patients undergoing TEE-guided tricuspid transcatheter edge-to-edge repair, Kuecken et al. identified new esophageal or gastric lesions in 24 patients (60%), with eight patients (20%) receiving endoscopic clip treatment [13]. These findings demonstrate that endoscopically detected injury is not confined to a single structural-heart cohort. Nevertheless, substantial differences in patient populations, procedural TEE exposure, and lesion definitions prevent direct comparison of these rates or extrapolation to LAAO.

The LAAO-specific evidence is substantially more limited. Afzal et al. reported at least one TEE-related adverse event in 11 of 229 patients (4.8%) undergoing LAA closure, but the composite included bleeding, mechanical complications, conversion to endotracheal intubation, and pneumonia [18]. Notably, no esophageal lesion or dysphagia was documented in the LAA subgroup, although six patients experienced bleeding complications, including two cases of hematemesis [18]. These findings are reassuring with respect to clinically recognized mechanical esophageal injury but do not exclude subclinical mucosal trauma because routine post-procedural endoscopy was not performed. Similarly, the SOLOCLOSE series reported a single immediate esophageal trauma among 208 consecutive patients undergoing TEE-guided LAAO [19]. These studies provide valuable procedure-specific data but were not designed primarily to detect the complete spectrum of esophageal mucosal injury.

Additional LAAO-specific information is available from a large retrospective electronic health record analysis by Hasnie et al. Among 3042 patients undergoing TEE-guided LAAO, 262 (8.6%) experienced a major complication within 72 h, with gastrointestinal hemorrhage accounting for 250 of these events (95.4%) [29]. However, the composite outcome included gastrointestinal bleeding, esophageal injury, and upper respiratory tract injury identified through administrative diagnostic codes. The database did not reliably distinguish upper from lower gastrointestinal bleeding or establish whether bleeding was directly caused by TEE-related mucosal trauma. Furthermore, the procedural subgroup could be identified for only a proportion of the overall study population. Consequently, the reported 8.6% represents the frequency of a broad coded complication composite in the LAAO subgroup and should not be interpreted as the incidence of TEE-associated esophageal injury.

A complementary perspective is provided by Zahid et al., who analyzed 61,995 weighted LAA closure hospitalizations in the US National Inpatient Sample. In their propensity-matched comparison, gastrointestinal bleeding was more frequently recorded following TEE-guided than ICE-guided procedures (3.5% vs. 2.1%; OR for ICE, 0.59; 95% CI 0.37–0.94) [30]. Although these findings suggest a potentially relevant difference in gastrointestinal bleeding outcomes between imaging strategies, the administrative nature of the data, lack of anatomical localization of bleeding, and potential residual confounding prevent attribution of this difference specifically to esophageal injury caused by TEE.

Additional LAAO-specific evidence is provided by Darden et al., who analyzed 49,357 patients undergoing LAAO in the National Cardiovascular Data Registry Left Atrial Appendage Occlusion Registry. In-hospital gastrointestinal bleeding was reported in 92 patients (0.19%), while esophageal injury was recorded in 51 patients (0.10%) [31]. Although these findings provide procedure-specific estimates of clinically reported complications, the registry-based design did not establish whether esophageal injuries or gastrointestinal bleeding were directly attributable to TEE instrumentation.

The reported frequency of gastrointestinal bleeding varies markedly across the three largest LAAO-specific datasets, ranging from 0.19% in the NCDR registry reported by Darden et al. to 3.5% among TEE-guided procedures in the National Inpatient Sample analysis by Zahid et al. and 8.2% in the LAAO subgroup analyzed by Hasnie et al. [29,30,31]. This substantial variation likely reflects differences in outcome definitions, registry-based versus administrative-code ascertainment, observation windows (in-hospital versus a fixed 72 h period), and the inclusion of gastrointestinal bleeding without consistent anatomical localization. Consequently, these rates are not directly comparable and should not be interpreted as estimates of TEE-associated esophageal injury.

This distinction has important methodological implications. Available data do not support calculation of a robust pooled incidence of esophageal injury specifically attributable to TEE-guided LAAO. Studies differ in procedural setting, outcome definition, intensity of post-procedural surveillance, anesthesia strategy, and whether gastrointestinal events were actively sought or recorded only when clinically apparent [9,10,18,29]. Furthermore, most detailed evidence regarding severe esophageal complications comes from case reports, which demonstrate the spectrum and potential severity of injury but cannot establish frequency or comparative risk [11,20,21]. The available LAAO-specific evidence on TEE-associated esophageal and gastrointestinal complications is summarized in Table 1.

### 3.2. Mechanisms of Esophageal Injury

TEE-related injury is likely multifactorial. Direct mechanical trauma may occur during probe insertion, advancement, flexion, rotation, or repeated repositioning, whereas sustained contact between the probe and the esophageal wall may result in focal pressure injury during prolonged procedures [6,7,8,10,32]. The prospective observations of Freitas-Ferraz et al. provide particularly relevant mechanistic evidence. In the prospective study by Freitas-Ferraz et al., each additional 10 min of procedural time under TEE manipulation, defined by the investigators as imaging time, was independently associated with higher odds of complex esophageal or gastric lesions (OR 1.27, 95% CI 1.01–1.59). Poor or suboptimal image quality was also an independent predictor (OR 4.93, 95% CI 1.10–22.02) [10].

However, the available evidence regarding the relationship between procedural duration and mucosal injury remains inconclusive. In the prospective study by Freitas-Ferraz et al., involving 50 patients, longer procedural time under TEE manipulation (imaging time) was associated with increased odds of complex esophageal or gastric lesions, although the confidence interval was relatively wide and its lower bound was close to unity (OR 1.27 per 10 min increment, 95% CI 1.01–1.59) [10]. In contrast, Kuecken et al. found no statistically significant differences in procedure duration or the number of TEE probe positional changes between patients with and without endoscopically detected lesions in a cohort of 40 patients [13]. Given the small sample sizes, differences in procedural settings and exposure measurements, neither study provides definitive evidence for or against an association between TEE duration and mucosal injury. Larger prospective studies using standardized measures of TEE exposure are needed to clarify this relationship.

Difficult imaging may require repeated acquisition of views and more extensive manipulation or flexion of the probe, providing a plausible mechanism for increased cumulative mechanical stress. Reduced tissue perfusion may theoretically increase susceptibility to pressure-related mucosal injury during prolonged TEE exposure. However, direct evidence supporting esophageal hypoperfusion as an independent mechanism of injury during LAAO is lacking, and this possibility should remain speculative. These considerations are especially pertinent to LAAO because TEE is used dynamically throughout several procedural stages rather than as a brief diagnostic examination. Transseptal puncture, LAA sizing, device positioning, assessment of compression and stability, evaluation of peri-device leak, and exclusion of procedural complications may all require repeated acquisition of different imaging planes [2,3,5]. Procedure duration and actual probe manipulation time, however, should not be regarded as interchangeable, and future studies should distinguish these variables whenever possible. Thermal injury has also been discussed as a potential mechanism in the broader TEE literature [6,8], although its relative contribution during LAAO remains uncertain. Accordingly, prolonged TEE exposure is probably best viewed as a composite marker of cumulative mechanical and physiological stress rather than as evidence for a single injury mechanism.

### 3.3. Patient Vulnerability and Antithrombotic Exposure

Patient-related susceptibility is likely to interact with procedural factors. Individuals referred for LAAO are often elderly and have multiple comorbidities, while many have a history of clinically important bleeding or difficulty maintaining long-term anticoagulation [1,2,17].

In the prospective study by Freitas-Ferraz et al., abnormal baseline esophagogastroduodenoscopy findings were more frequent among patients who subsequently developed complex esophageal or gastric lesions than among those without complex lesions (70% vs. 37%; *p* = 0.04). However, baseline endoscopic abnormalities were not identified as independent predictors in the reported multivariable analysis [10]. These findings suggest a possible relationship between pre-existing upper gastrointestinal pathology and susceptibility to TEE-associated injury, although an independent contribution has not been established.

Additional evidence regarding patient-related vulnerability comes from the retrospective study by Freitas-Ferraz et al., which included 1249 patients undergoing TEE-guided interventional cardiology procedures [32]. The investigators reported a higher frequency of major TEE-related complications in the subgroup undergoing interventions requiring more extensive TEE guidance, including LAAO, mitral transcatheter edge-to-edge repair, and paravalvular leak closure, compared with patients undergoing TAVI (2.8% vs. 0.6%). Factors associated with increased complication risk included low body weight, a previous history of gastrointestinal bleeding, and chronic corticosteroid or immunosuppressive therapy. Furthermore, longer procedural time under TEE manipulation was independently associated with major complications (OR 1.13 per 10-min increment in imaging time, 95% CI 1.01–1.25) [32]. These findings are particularly relevant because previous gastrointestinal bleeding is a frequent clinical consideration in patients referred for LAAO. Nevertheless, the identified associations were derived from a mixed interventional population and should not be interpreted as independent predictors of esophageal injury specifically during LAAO. In the study by Afzal et al., lower baseline hemoglobin levels and obstructive sleep apnea were associated with TEE-related adverse events during structural heart interventions [18]. These findings further suggest that patient-related vulnerability may extend beyond pre-existing esophageal disease. However, the reported associations concern a broad adverse-event composite and should not be interpreted as established predictors of esophageal injury specifically during LAAO.

Observational data in patients with pre-existing esophageal conditions, including esophageal stricture, previous esophageal surgery, esophageal varices, and altered upper gastrointestinal anatomy after bariatric surgery, further support the importance of individualized assessment before TEE [33,34,35,36].

Patient-level analyses from the broader structural-heart cohort reported by Hasnie et al. identified higher frequencies of the composite major complication outcome among patients aged 65 years or older and those receiving anticoagulant or antiplatelet therapy [29]. These associations are clinically relevant to the LAAO population, although they were not established specifically for esophageal injury or independently validated within the LAAO subgroup. The predominance of gastrointestinal bleeding among recorded LAAO complications further emphasizes the need to distinguish underlying hemorrhagic vulnerability from direct mechanical injury caused by TEE.

Antithrombotic therapy may be particularly important in determining the clinical expression of an injury. Intraprocedural anticoagulation is required during LAAO, and post-procedural antithrombotic therapy remains necessary in most patients, although contemporary regimens vary according to device, thrombotic risk, bleeding history, and institutional practice [2,17]. Anticoagulation does not necessarily initiate mechanical mucosal injury, but it may increase the extent of bleeding once tissue disruption has occurred. The case reported by Cheng et al. illustrates this potential interaction. The patient underwent preprocedural TEE followed by TEE-guided LAAO with substantial intraprocedural heparin administration and subsequently developed an extensive submucosal esophageal hematoma [20]. Although repeated esophageal instrumentation and anticoagulation may have contributed to the development or severity of the hematoma, their individual causal contributions cannot be established from a single case report.

### 3.4. Clinical Spectrum, Diagnosis, and Management

The clinical spectrum extends from asymptomatic mucosal abnormalities to potentially life-threatening structural injury. While clinically reported TEE-associated gastrointestinal complications are relatively uncommon [9], studies employing systematic endoscopic assessment have identified minor mucosal abnormalities, including petechiae, ecchymoses, superficial abrasions, and small lacerations, which may have little immediate clinical consequence and remain unrecognized in routine clinical practice [10,12,13].

At the other extreme, intramural hematoma, major gastrointestinal bleeding, perforation, and fistula formation can produce substantial morbidity [8,11,20,21,37,38].

The recent report of an esophageal–pleural fistula following TEE-guided LAAO is particularly instructive because the presentation was not initially dominated by typical gastrointestinal symptoms. A 77-year-old woman developed pneumothorax and persistent pleural effusion, and the diagnosis was ultimately established after food particles were identified in chest-tube drainage [21]. The procedure was performed under deep sedation without endotracheal intubation, and difficult TEE probe insertion was reported. The patient also had a history of gastrointestinal bleeding and was receiving antiplatelet therapy [21]. These features illustrate the potential coexistence of procedural difficulty and patient-related bleeding vulnerability, although their individual contributions to fistula formation cannot be established.

This case expands the recognized clinical spectrum and illustrates that delayed or atypical thoracic manifestations may occasionally reflect occult esophageal injury.

Following TEE-guided LAAO, new dysphagia, odynophagia, retrosternal or chest pain, hematemesis, unexplained hemoglobin decline, or evidence of upper gastrointestinal bleeding should therefore prompt consideration of an esophageal complication. Persistent pleural effusion, pneumothorax, systemic inflammatory signs, or otherwise unexplained thoracic findings may warrant similar consideration when the clinical course is atypical [21,37,38]. The diagnostic approach should be individualized according to clinical stability and the suspected lesion. Cross-sectional imaging is particularly useful when deep mural injury, perforation, or fistula is suspected, whereas endoscopy permits direct assessment of mucosal pathology but requires appropriate caution when major mural disruption or perforation is suspected [8,38].

Management likewise depends on the type and severity of injury. Minor mucosal lesions are generally managed conservatively. More substantial injuries may require temporary fasting, nutritional support, acid suppression, modification of antithrombotic therapy, close clinical and radiological surveillance, and antimicrobial therapy when perforation or infection is present or suspected [8,20,21,39]. Endoscopic or surgical intervention may be required in selected cases [38,40]. However, the very small number of LAAO-specific severe injuries precludes development of a dedicated evidence-based management algorithm.

### 3.5. Prevention and Pre-Procedural Risk Assessment

The limited evidence base does not support routine pre-procedural upper endoscopy for all patients scheduled for LAAO. Nevertheless, careful history taking is appropriate, with particular attention to dysphagia, odynophagia, previous esophageal surgery, known strictures or diverticula, significant esophagitis, previous upper gastrointestinal bleeding, and other established structural abnormalities [6,7,8,33,34,35,36]. When clinically significant esophageal disease is suspected, targeted gastroenterological assessment may be reasonable before selecting the intraprocedural imaging strategy [8,33,34]. The role of pharmacological prophylaxis is less well defined. Proton pump inhibitors may be considered for symptomatic management of upper gastrointestinal mucosal injury following TEE, although evidence supporting their effectiveness specifically for mechanically induced esophageal injury remains limited [10,29].

However, evidence supporting routine prophylactic acid suppression specifically for the prevention of mechanically mediated TEE-associated esophageal injury during LAAO is currently lacking. Therefore, proton pump inhibitor therapy should not be considered a substitute for careful patient selection and minimization of unnecessary probe manipulation.

Procedural prevention is largely based on mechanistic reasoning and observational evidence. Minimizing unnecessary probe manipulation, avoiding excessive flexion, limiting prolonged fixed probe positions, and obtaining the imaging required for safe device implantation with the least unnecessary manipulation are rational strategies [6,7,8,10,32]. The association between TEE manipulation time and complex lesions identified by Freitas-Ferraz et al. provides observational support for reducing unnecessary exposure [10]. However, these strategies have not been prospectively tested specifically as interventions for preventing esophageal complications during LAAO.

Importantly, procedural efficiency should not come at the expense of adequate imaging. The objective should not be to minimize TEE duration indiscriminately but to avoid unnecessary manipulation while preserving the imaging required for safe transseptal access, device sizing and deployment, assessment of device stability, and recognition of immediate procedural complications [2,3,5,41].

#### ICE and Alternative Imaging Strategies

The increasing use of ICE provides a clinically relevant alternative in patients in whom esophageal instrumentation is undesirable. By eliminating passage and manipulation of an esophageal probe, ICE removes the direct mechanism responsible for TEE-related esophageal trauma and may facilitate LAAO without general anesthesia in appropriate settings [25,26,42,43,44].

However, available comparative evidence does not support describing ICE as universally safer than TEE. In a meta-analysis of 19 observational studies involving 42,474 patients, Serpa et al. reported similar overall complication rates between ICE- and TEE-guided LAAO, although pericardial effusion and residual iatrogenic atrial septal defect were more frequent with ICE [22]. Beneki et al., analyzing 18 studies involving 124,230 patients, found fewer overall bleeding events with ICE guidance, whereas major bleeding did not differ significantly between the two modalities. Conversely, ICE was associated with higher rates of pericardial effusion or tamponade, residual iatrogenic atrial septal defect, and vascular complications [23]. More recently, Yaser et al. included 27 observational studies and reported comparable procedural success between ICE and TEE, but higher odds of pericardial effusion requiring intervention (adjusted OR 1.74, 95% CI 1.15–2.64) and residual iatrogenic atrial septal defect (unadjusted OR 1.61, 95% CI 1.20–2.16) with ICE guidance [24]. Importantly, these meta-analyses did not provide pooled estimates of esophageal injury or anatomically defined upper gastrointestinal complications. Therefore, although ICE eliminates the need for esophageal probe insertion, the magnitude of any reduction in clinically documented esophageal injury during LAAO remains unquantified. Miniaturized TEE represents an additional imaging strategy that may facilitate LAAO under conscious sedation without routine general anesthesia. In a multicenter observational study of 546 consecutive LAAO procedures performed using miniaturized TEE guidance, Aminian et al. reported a technical success rate of 98.0%, with conversion to general anesthesia required in only 0.7% of patients [45]. These findings support the feasibility of miniaturized TEE as an alternative to conventional TEE guidance in selected patients. However, miniaturized probes still require esophageal instrumentation, and the available evidence does not establish whether their use reduces endoscopically confirmed esophageal injury. Furthermore, the potential advantages of conscious sedation should be distinguished from any presumed reduction in mechanical esophageal trauma, as these represent different clinical outcomes.

Thus, the clinically relevant question is not whether ICE is globally safer than TEE but whether avoiding esophageal instrumentation provides a meaningful advantage in an individual patient whose esophageal risk is increased. Known esophageal disease, previous esophageal intervention, difficult prior TEE, significant dysphagia, or other factors suggesting increased susceptibility to mechanical injury may shift the balance toward ICE when adequate expertise is available [5,43,44]. Conversely, cardiac anatomy, vascular access, operator experience, institutional expertise, and the distinct complication profile of ICE remain important considerations [22,23,24,43].

CCT further expands the multimodality approach by providing a detailed anatomical assessment before LAAO and an increasingly used option for post-procedural surveillance [2,46,47,48,49,50,51]. CCT does not generally provide the continuous real-time guidance required during device implantation, but greater integration of CCT for planning and follow-up may reduce cumulative exposure to TEE [46,47,48,49,51]. Contemporary LAAO imaging may therefore increasingly involve complementary use of CCT, ICE, and TEE according to individual patient characteristics rather than reliance on a single modality throughout the entire procedural pathway [3,4,5,45,52].

The principal characteristics of TEE, ICE, and CCT in the context of increased esophageal risk are summarized in Table 2.

## 4. Limitations of the Current Evidence and Future Directions

The most important limitation of the available literature is the scarcity of studies specifically designed to evaluate esophageal injury during LAAO. Much of the mechanistic and epidemiological evidence derives from mixed structural-heart populations in which LAAO represents only one procedural subgroup [10,18,29,32]. Such populations differ in procedural duration, intensity of probe manipulation, anesthesia, anticoagulation, and baseline comorbidity, limiting direct extrapolation to LAAO. In contrast to previous reviews primarily addressing procedural imaging outcomes or TEE-related complications across heterogeneous cardiac interventions, the present synthesis specifically highlights the absence of standardized esophageal injury endpoints in LAAO studies and the resulting limitations in attributing gastrointestinal events to TEE instrumentation [27,28]. A second limitation is substantial heterogeneity in outcome definitions. Most available studies rely on clinically recorded gastrointestinal bleeding, mechanical injury, dysphagia, or broader TEE-related adverse-event composites [9,18,29,32]. In contrast, a smaller number of investigations have used systematic endoscopic assessment to identify mucosal lesions that might otherwise remain clinically unrecognized [10,12,13].

Consequently, the absence of a reported esophageal complication does not necessarily indicate the absence of injury. Conversely, minor lesions identified by systematic endoscopy should not automatically be considered clinically meaningful complications.

Third, severe LAAO-specific injuries are represented predominantly by isolated case reports [20,21]. These reports are valuable for defining possible presentations, diagnostic pathways, and management strategies but are inherently vulnerable to publication bias and cannot establish incidence or independent risk factors [11,37,38,39].

Future prospective LAAO registries should therefore incorporate standardized definitions of TEE-associated gastrointestinal injury and separately report clinically relevant esophageal events. Relevant variables should include baseline esophageal disease and symptoms, previous gastrointestinal bleeding, anesthesia strategy, duration of active TEE manipulation, difficulty of probe insertion or imaging, intraprocedural anticoagulation, post-procedural antithrombotic therapy, post-procedural gastrointestinal symptoms, diagnostic investigations, treatment, and clinical outcome. Comparative studies of TEE and ICE should specifically capture esophageal and upper gastrointestinal endpoints rather than subsuming them within broad procedural or bleeding composites [5,22,23,24,43].

Ultimately, development of a clinically useful risk-stratification approach may permit identification of patients in whom the advantages of avoiding esophageal instrumentation outweigh the distinct procedural risks and logistical requirements of alternative imaging modalities [3,5,44].

## 5. Conclusions

TEE remains an effective and widely used imaging modality for guiding percutaneous LAAO, but clinically significant esophageal injury represents an uncommon and potentially underrecognized component of its safety profile [8,9,10]. The available literature does not permit a reliable estimate of the incidence of esophageal injury specifically attributable to TEE-guided LAAO. LAAO-specific observational data remain limited, while much of the evidence regarding injury mechanisms, risk factors, and the broader spectrum of mucosal damage derives from heterogeneous structural-heart and general TEE populations [18,19,29,32].

Available evidence suggests that TEE-associated esophageal injury exists along a continuum from clinically silent mucosal abnormalities to rare but potentially severe complications, including intramural hematoma, major bleeding, perforation, and fistula formation [9,11,20,21,32]. Mechanical trauma and prolonged probe manipulation appear central to pathogenesis, while pre-existing esophagogastric abnormalities, advanced age, and antithrombotic exposure may influence susceptibility or clinical expression of injury [6,10,29,33,34]. However, the relative contribution of these factors specifically during LAAO remains insufficiently defined.

Recognition of this risk should not discourage appropriate use of TEE. Rather, it supports careful pre-procedural assessment, attention to symptoms or known esophageal pathology, judicious probe manipulation, and early investigation of compatible post-procedural symptoms or otherwise unexplained thoracic findings [7,8]. ICE eliminates esophageal instrumentation and therefore represents an attractive alternative in selected patients at increased esophageal risk, but its distinct complications preclude characterization as universally safer than TEE [22,23,24,43]. Increasing integration of cardiac CT may further reduce cumulative reliance on TEE during pre- and post-procedural assessment [46,48,49,51].

Ultimately, imaging for LAAO should be individualized according to patient characteristics, esophageal risk, procedural anatomy, anesthesia considerations, operator expertise, and institutional experience [3,4,5]. Prospective studies using standardized definitions and dedicated assessment of esophageal outcomes are required to determine the true burden of TEE-associated injury during LAAO and to establish which patients may derive the greatest clinical benefit from alternative imaging strategies.

## Figures and Tables

**Table 1:** LAAO-specific evidence on TEE-associated esophageal and gastrointestinal complications.

Study	LAAO Population	Ascertainment and Endpoint	Findings
Afzal et al. (2022) [18]	Retrospective, single-center structural-heart cohort; separate LAA closure subgroup, *n* = 229 (of 898 procedures); TEE under procedural sedation.	Clinically documented TEE-related adverse-event composite (bleeding, mechanical injury, intubation conversion, pneumonia); individual bleeding and esophageal symptoms; no routine postprocedural endoscopy.	*n* = 11/229 (4.8%) had ≥1 composite event. Six had bleeding complications, including two with hematemesis. No esophageal lesion or dysphagia was documented in the LAA closure subgroup. The composite is not an esophageal injury rate.
Golzarian et al. (2025), SOLOCLOSE [19]	Prospective, single-center series; *n* = 208 consecutive patients undergoing single-operator TEE-guided LAAO; 200 technically successful procedures.	Clinically recorded immediate procedural complications, including esophageal trauma; major bleeding assessed through 45 days; no systematic endoscopic screening reported.	One immediate esophageal trauma (*n* = 1/208; 0.5%). Five major bleeding events at 45 days (*n* = 5/200; 2.5% of technically successful cases in reported analysis), not identified as esophageal or gastrointestinal bleeding.
Hasnie et al. (2023) [29]	Retrospective electronic-health-record study of TEE-guided structural interventions; separately identifiable LAAO subgroup, *n* = 3042.	Administrative code ascertainment within 72 h; composite of gastrointestinal hemorrhage, esophageal injury, and upper respiratory tract injury; no endoscopic adjudication or localization of GI bleeding.	*n* = 262/3042 (8.6%) met the broad composite; *n* = 250/262 (95.4%) were coded as GI hemorrhage (*n* = 250/3042, 8.2%). Anatomical source and causation by TEE could not be determined; 8.6% is not the esophageal-injury incidence.
Zahid et al. (2022) [30]	US National Inpatient Sample, *n* = 61,995 weighted LAA closure hospitalizations (TEE-guided 60,585; ICE-guided 1410); propensity-matched imaging comparison.	In-hospital coded gastrointestinal bleeding, compared between TEE and ICE; no lesion-specific endoscopic verification; no attribution of bleeding source to probe trauma.	GI bleeding: 3.5% with TEE versus 2.1% with ICE (OR for ICE 0.59; 95% CI 0.37–0.94). Retroperitoneal bleeding was more frequent with ICE (0.7% vs. 0%). These findings indicate different bleeding profiles but do not establish TEE-induced esophageal injury.
Darden et al. (2021) [31]	Retrospective NCDR LAAO Registry study; *n* = 49,357 patients undergoing WATCHMAN LAAO (20,388 women and 28,969 men).	Registry-recorded in-hospital gastrointestinal bleeding and esophageal injury, stratified by sex. No routine endoscopic screening or TEE-specific causal adjudication.	In-hospital GI bleeding was reported in 92/49,357 patients (0.19%), while esophageal injury was recorded in 51/49,357 patients (0.10%). Neither endpoint was proven attributable to TEE instrumentation.
Cheng et al. (2022) [20]	Single patient, 70-year-old man undergoing TEE-guided WATCHMAN LAAO; preprocedural and intraprocedural TEE; intraprocedural heparin.	Symptom-triggered chest CT followed by upper gastrointestinal endoscopy confirming an extensive esophageal hematoma and mucosal exfoliation. Follow-up endoscopy documented complete resolution. No routine endoscopic screening.	Extensive submucosal esophageal hematoma following TEE-guided LAAO, initially presenting with severe retrosternal pain and odynophagia, followed by hematemesis, hematochezia, and a marked decrease in hemoglobin. The hematoma resolved with conservative management. This case demonstrates severe anatomically confirmed injury but cannot establish incidence or independent causation.
Qiu et al. (2026) [21]	Single patient, 77-year-old woman undergoing TEE-guided LAAO under deep sedation; difficult probe insertion reported.	Clinical investigation of pneumothorax and persistent pleural effusion; food particles in chest-tube drainage and follow-up imaging confirmed esophageal–pleural fistula.	Esophageal–pleural fistula with atypical thoracic presentation; closed after 52 days of conservative management. A case report cannot quantify risk or isolate the contribution of TEE.

Abbreviations: CT, computed tomography; GI, gastrointestinal; ICE, intracardiac echocardiography; LAAO, left atrial appendage occlusion; TEE, transesophageal echocardiography. Note: Only separately identifiable LAAO outcomes are included. Clinical events, coded bleeding, and anatomically confirmed esophageal injury are distinct endpoints and must not be pooled. Mixed cohorts without LAAO-specific results are excluded. SOLOCLOSE major bleeding is contextual, not a TEE-associated GI injury endpoint.

**Table 2:** Comparison of imaging strategies for LAAO in patients with increased esophageal risk.

Characteristics	TEE	ICE	CCT
Primary role in LAAO	Established real-time intraprocedural guidance.	Real-time intraprocedural guidance without an esophageal probe.	Anatomical assessment for preprocedural planning and postprocedural surveillance.
Esophageal instrumentation	Required; probe insertion and manipulation expose the esophagus to mechanical stress.	Not required; avoids direct TEE-related probe trauma.	Not required; avoids esophageal probe exposure.
Intraprocedural visualization	Real-time multiplanar imaging for transseptal puncture, LAA sizing, device deployment, stability, and peri-device leak assessment.	Real-time guidance for transseptal access, LAA assessment and device deployment; imaging depends on catheter positioning and operator experience.	Does not generally provide continuous real-time guidance during device implantation.
Anesthesia and procedural considerations	Conventional TEE is frequently performed under general anesthesia or deep sedation; practices vary.	May allow LAAO without general anesthesia in appropriately selected settings.	Noninvasive CT acquisition; not a substitute for intraprocedural imaging guidance.
Relevance to increased esophageal risk	Less attractive when significant esophageal disease, dysphagia, prior esophageal intervention, or difficult previous TEE raises concern about probe trauma.	Particularly relevant when avoiding esophageal instrumentation is desirable and appropriate expertise is available.	Useful for planning and follow-up while reducing cumulative TEE exposure; does not replace intraprocedural guidance.
Important limitations or risks	Esophageal/gastric trauma and GI bleeding may occur; LAAO-specific incidence of TEE-attributable esophageal injury is uncertain.	Distinct risks include vascular complications, pericardial effusion/tamponade, and residual iatrogenic atrial septal defect in some comparative datasets.	Radiation and iodinated contrast exposure; image quality may be affected by rhythm and other technical factors.
Evidence and interpretation	Broad TEE and mixed structural-heart data cannot be used as LAAO-specific esophageal injury rates; direct LAAO data remain limited.	Comparative LAAO studies show broadly similar technical success to TEE but do not quantify a reduction in esophageal injury.	Supports pre-/postprocedural anatomical assessment; reduction in cumulative TEE exposure is plausible, not a proven reduction in clinical esophageal events.
Practical selection	Consider when esophageal access is acceptable and high-quality real-time TEE is needed.	Consider when esophageal instrumentation should be avoided, accounting for expertise and procedural risk.	Integrate with TEE or ICE for individualized preprocedural planning and follow-up.

Abbreviations: CCT, cardiac computed tomography; CT, computed tomography; GI, gastrointestinal; ICE, intracardiac echocardiography; LAAO, left atrial appendage occlusion; LAA, left atrial appendage; TEE, transesophageal echocardiography. Note: Modality selection should be individualized. Avoiding esophageal instrumentation eliminates direct probe-related exposure but does not eliminate other procedure-related risks. CCT is complementary to, rather than a replacement for, real-time intraprocedural guidance.

## Data Availability

No new data were created or analyzed in this study. Data sharing is not applicable to this article.

## Abbreviations

The following abbreviations are used in this manuscript:

Abbreviation	Definition
AF	Atrial fibrillation
CCT	Cardiac computed tomography
EGD	Esophagogastroduodenoscopy
ICE	Intracardiac echocardiography
LAAO	Left atrial appendage occlusion
TEE	Transesophageal echocardiography
TOE	Transoesophageal echocardiography
TAVI	Transcatheter aortic valve implantation
